# Microbial Diversity and Mercury Methylation Activity in Periphytic Biofilms at a Run-of-River Hydroelectric Dam and Constructed Wetlands

**DOI:** 10.1128/mSphere.00021-21

**Published:** 2021-03-17

**Authors:** Maxime Leclerc, Makayla C. Harrison, Veronika Storck, Dolors Planas, Marc Amyot, David A. Walsh

**Affiliations:** a GRIL, Département de Sciences Biologiques, Université de Montréal, Montréal, Québec, Canada; b Department of Biology, Concordia University, Montréal, Québec, Canada; c GRIL, Département de Sciences Biologiques, Université du Québec à Montréal, Montréal, Québec, Canada; University of Iowa

**Keywords:** methylmercury, mercury, periphyton, *hgcAB*, freshwater, wetlands, metagenomics

## Abstract

Periphytic biofilms have the potential to greatly influence the microbial production of the neurotoxicant monomethylmercury in freshwaters although few studies have simultaneously assessed periphyton mercury methylation and demethylation rates and the microbial communities associated with these transformations. We performed a field study on periphyton from a river affected by run-of-river power plants and artificial wetlands in a boreal landscape (Québec, Canada). *In situ* incubations were performed on three sites using environmental concentrations of isotopically enriched monomethylmercury (MM^198^Hg) and inorganic mercury (^200^Hg) for demethylation and methylation rate measurements. Periphytic microbial communities were investigated through 16S rRNA gene analyses and metagenomic screenings for the *hgcA* gene, involved in mercury methylation. Positive mercury methylation rates ([5.9 ± 3.4] × 10^−3^ day^−1^) were observed only in the wetlands, and demethylation rates averaged 1.78 ± 0.21 day^−1^ for the three studied sites. The 16S rRNA gene analyses revealed *Proteobacteria* as the most abundant phylum across all sites (36.3% ± 1.4%), from which families associated with mercury methylation were mostly found in the wetland site. Metagenome screening for HgcA identified 24 different *hgcA* sequences in the constructed wetland site only, associated with 8 known families, where the iron-reducing *Geobacteraceae* were the most abundant. This work brings new information on mercury methylation in periphyton from habitats of impacted rivers, associating it mostly with putative iron-reducing bacteria.

**IMPORTANCE** Monomethylmercury (MMHg) is a biomagnifiable neurotoxin of global concern with risks to human health mostly associated with fish consumption. Hydroelectric reservoirs are known to be sources of MMHg many years after their impoundment. Little is known, however, on run-of-river dams flooding smaller terrestrial areas, although their numbers are expected to increase considerably worldwide in decades to come. Production of MMHg is associated mostly with anaerobic processes, but Hg methylation has been shown to occur in periphytic biofilms located in oxic zones of the water column. Therefore, in this study, we investigated *in situ* production of MMHg by periphytic communities in habitats impacted by the construction of a run-of-river dam by combining transformation rate measurements with genomic approaches targeting *hgcAB* genes, responsible for mercury methylation. These results provide extended knowledge on mercury methylators in river ecosystems impacted by run-of-river dams in temperate habitats.

## INTRODUCTION

The principal input of mercury (Hg) from watersheds to boreal lakes and rivers is through atmospheric deposition and anthropogenic perturbations (1). Once in aquatic systems, local methylation by anaerobic microbial processes produces monomethylmercury (MMHg), which is a potent neurotoxin. MMHg can be exported to the water column by diffusion (2), where it can undergo biomagnification through aquatic food chains (3). Anoxic sediments have long been considered the primary location of MMHg production (4), but their central role in MMHg production has been recently challenged by studies on Hg transformations in littoral biofilms (i.e., periphyton) (5, 6), which consist of heterogeneous communities of photosynthetic and heterotrophic microorganisms attached to a submerged substrate (7). Periphyton was previously shown to be an important site of Hg accumulation (8–10) and a source of Hg for entry into benthic trophic webs (11, 12). However, knowledge gaps remain on the contribution of periphyton to Hg cycling in boreal aquatic environments, specifically on the capacity of periphyton to produce MMHg.

A broad diversity of microbial groups is implicated in Hg methylation. Traditionally, sulfate-reducing bacteria (SRB) were considered the predominant Hg-methylating metabolic guild (13, 14). More recently, a combination of methylation assays using metabolic inhibitors and molecular genetic surveys of microbial assemblages broadened the diversity of putative Hg methylators to include iron-reducing bacteria (FeRB) (15) and methanogens (16). The discovery of the genetic basis for Hg methylation (*hgcA* and *hgcB* genes that encode a corrinoid iron-sulfur protein and a ferredoxin protein, respectively) (17) has facilitated studies that have expanded the diversity of putative Hg methylators and explored their distributions in the environment (18–22). Numerous additional bacterial phyla are now known to be implicated in MMHg production, including *Actinobacteria*, *Bacteroidetes*, *Firmicutes*, and *Chloroflexi* (17, 18, 23–25). The *hgcAB* genes were detected in periphyton from a high-altitude tropical lake (5) and subtropical wetlands of the Florida Everglades (26) with a predominance of sulfate reducers, methanogens, and syntrophs as putative Hg methylators. In addition to the genetic evidence of diverse methylator communities in periphyton, studies using isotopic approaches to estimate Hg transformation kinetics have shown that temperature, depth, light exposure, extracellular ligand concentrations, and biofilm structure are all factors influencing Hg methylation rates in periphyton (5, 6, 27–29).

Microbial processes also contribute to the demethylation of MMHg, producing inorganic mercury (IHg) in either its cationic (Hg^II+^) or elementary (Hg^0^) form. In sunlit waters, demethylation mainly occurs through abiotic photodegradation (30). However, in light-deficient aquatic environments, such as sediments or wetlands or at depth in the water column, MMHg demethylation is believed to occur mostly through microbial anaerobic oxidative or aerobic reductive demethylation (31). The *mer* operon, found in a wide variety of Hg-resistant microorganisms (32), is mainly associated with the aerobic reductive demethylation of Hg (31, 33). The oxidative demethylation is performed by anaerobic microorganisms lacking the *mer* operon that include SRB, FeRB, and methanogens also capable of Hg methylation (31). Hence, the combined activities of microbial methylation and demethylation contribute to the amount of MMHg available for biomagnification in aquatic food webs.

Dams associated with hydroelectric power plants in boreal ecosystems, particularly those with large reservoirs, are systems in which Hg methylation and food web transfer can occur for several decades after dam construction (34). The large-scale flooding of terrestrial soil leads to changes in redox conditions and microbial community composition that are conducive to MMHg production (35). As an alternative to large-reservoir hydroelectric dams, run-of-river hydroelectric dams are facilities that impound smaller terrestrial areas called pondage. Such run-of-river dams are assumed to have a reduced environmental footprint, but their environmental impact and their contribution to MMHg production are actually still largely unknown (36, 37). In these hydroelectric projects, wetlands are often constructed to compensate for the loss of habitat. Submerged trees and macrophytes in wetlands are colonized by microorganisms in the photic zone, potentially producing new sites of Hg methylation in periphytic biofilms (38). Although studies have been conducted on Hg methylation and demethylation in sediments of such altered systems (35), few have studied the contribution of periphytic communities to Hg cycling (37).

Here, we investigated the Hg methylation and demethylation capacity of periphyton communities by combining isotope-based *in situ* rate measurements with simultaneous assessment of periphyton diversity using 16S rRNA gene and metagenomic approaches targeting *hgcAB* genes. The study was performed on periphyton located at three sites upstream of a run-of-river hydroelectric plant on the St. Maurice River in Québec (Canada). These sites represented (i) a naturally submerged environment, (ii) a flooded environment due to dam construction, and (iii) an environment within a constructed wetland located upstream of the flooded area. Through comparison of Hg transformation rates and microbial community diversity, we assessed how environmental conditions at the three distinct locations, particularly the impact of flooding and wetland construction, influenced the development of Hg cycling communities. We hypothesized that (i) periphytic communities are heterogeneous between sampling sites, (ii) disturbed site communities have a greater abundance of Hg-methylating microorganisms, and (iii) communities enriched in putative microbial Hg-methylating taxa are associated with Hg methylation activities. Not many studies on Hg methylation by periphyton used natural undisturbed biofilms through *in situ* incubations (e.g., the work of S. Hamelin et al. [6] and W. L. Lazaro et al. [38]), as attempted in the present work, although several environmental and other factors related to the three-dimensional structure of biofilms have a major influence on Hg transformation rates. The results of this study contribute to the global efforts to extend the knowledge on Hg methylation in river ecosystems and the distribution of putative Hg methylators in general.

## RESULTS AND DISCUSSION

### Environmental context and Hg levels in water and periphytic biofilms.

**FIG 1:**
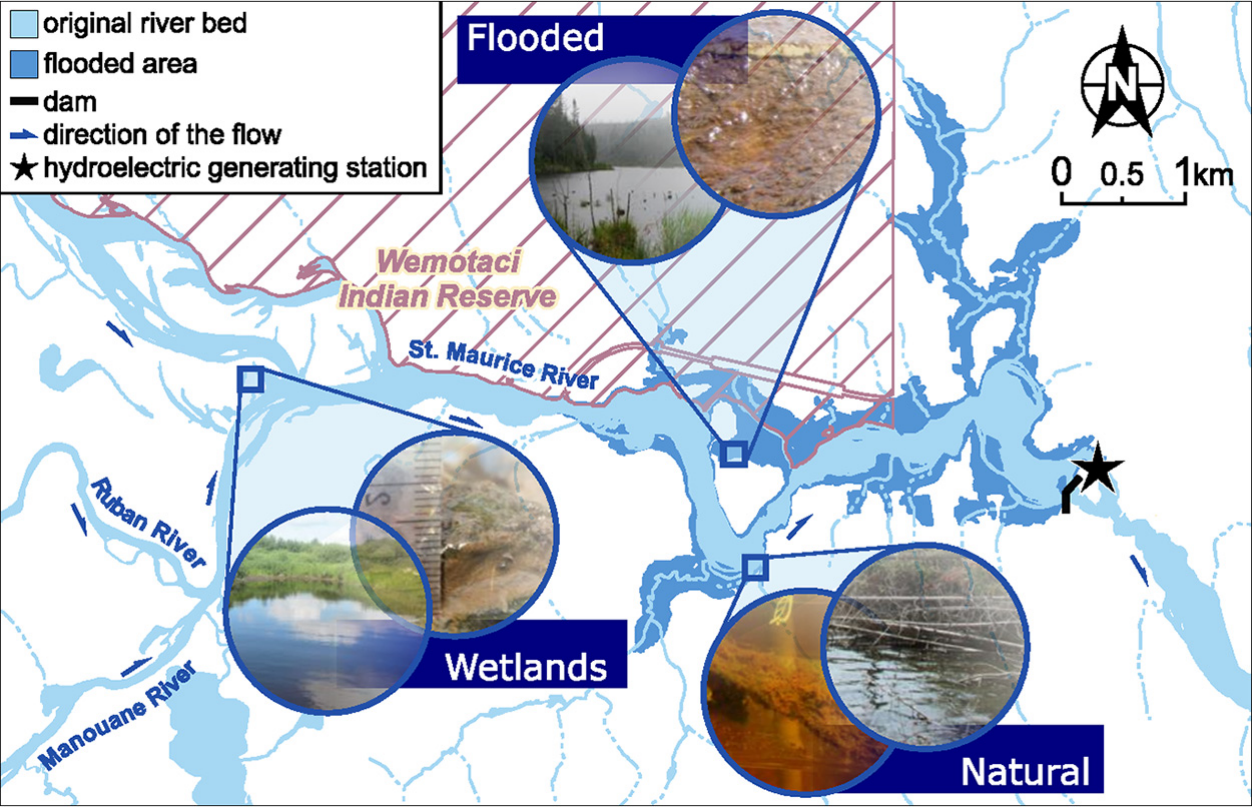
Location of experimental sampling sites on the St. Maurice River, with pictures of the natural, flooded, and constructed wetland sites with periphyton in the insets (map modified from Hydro-Québec).

Sampling sites upstream of the St. Maurice River Chute-Allard dam were selected to represent the different habitat types, which consisted of constructed wetlands, a flooded area, and naturally submerged habitat. Depth, water flow, and width differed between sites. The wetland site was the most stagnant and narrow, while the natural site was the deepest and had the highest flow rate. Dissolved oxygen concentrations ranged from 6.99 to 9.16 mg liter^−1^, and pH ranged between 5.2 and 6.0, with the lowest values for both parameters found in the wetland site (Table 1). Low oxygen and pH conditions in the wetland site are favorable for anaerobic MMHg production and also affect microbial activity and Hg speciation (39). The constructed wetlands were almost completely covered by macrophytes heavily colonized by thick periphytic biofilms (insets, Fig. 1). In contrast, the flooded and natural sites were essentially deprived of substrates for periphyton colonization in the water column except for a few scattered submerged wood stumps and a layer of sand on top of the sediments. Hence, periphyton from the wetlands is more likely to influence the surrounding water physicochemistry and MMHg levels due to high density and extensive coverage in comparison to the other sites.

**TABLE 1:** Water and periphyton chemistry of the studied sites

Variable	Natural	Flooded	Wetland
In water			
Depth (m)	3.2	1.4	1.4
pH	6.0	5.6	5.2
Redox potential (mV)	233	203	271
Dissolved oxygen (mg liter^−1^)	9.16	9.10	6.99
Dissolved organic carbon (mg C liter^−1^)	6.23 ± 0.21	6.17 ± 0.06	6.20 ± 0.10
Monomethylmercury (ng liter^−1^)	0.10 ± 0.01	0.12 ± 0.01	0.23 ± 0.02
Inorganic mercury (ng liter^−1^)	1.62 ± 0.30	1.25 ± 0.01	0.76 ± 0.04
In periphyton			
Monomethylmercury (ng g^−1^ of DW)	3.4	6.7	2.7
Inorganic mercury (ng g^−1^ of DW)	23.3	30.5	21.7
Ash-free dry wt/dry wt (%)	24.0	35.7 ± 0.6	26.3 ± 3.5

The highest MMHg concentration in water samples was measured in the constructed wetland site (23.2% of total Hg [THg]). In contrast to water samples, the highest MMHg concentration measured in periphyton was found at the flooded site (18.0% of THg), the lowest values were measured in the periphyton of the wetlands (11.2% of THg), and values for the natural site were in between (12.6% of THg). The ratios of MMHg to THg in periphyton have been reported to range from 0.1% to 36% but can exceed 75% in certain cases (10). Periphyton may be extensively heterogeneous from one site to another, and many environmental factors (e.g., light, nutrients, water flow, and nature of the substrate) that influence their growth and composition may explain this heterogeneity (40). The periphyton from the flooded site, which exhibited highest percent MMHg in periphyton, was richest in organic matter, as measured by the ash-free dry weight/dry weight (AFDW/DW) ratio (Table 1). Previous studies on periphyton showed relationships between Hg content and different indicators of organic matter such as the AFDW (41) or the autotrophic index, taking into account photosynthetic microorganisms (10). Organic matter can act on different steps of the Hg cycle by influencing its mobility, bioavailability, or the activity of methylating microorganisms, for instance (42).

### Mercury methylation and demethylation rates in periphyton.

**FIG 2:**
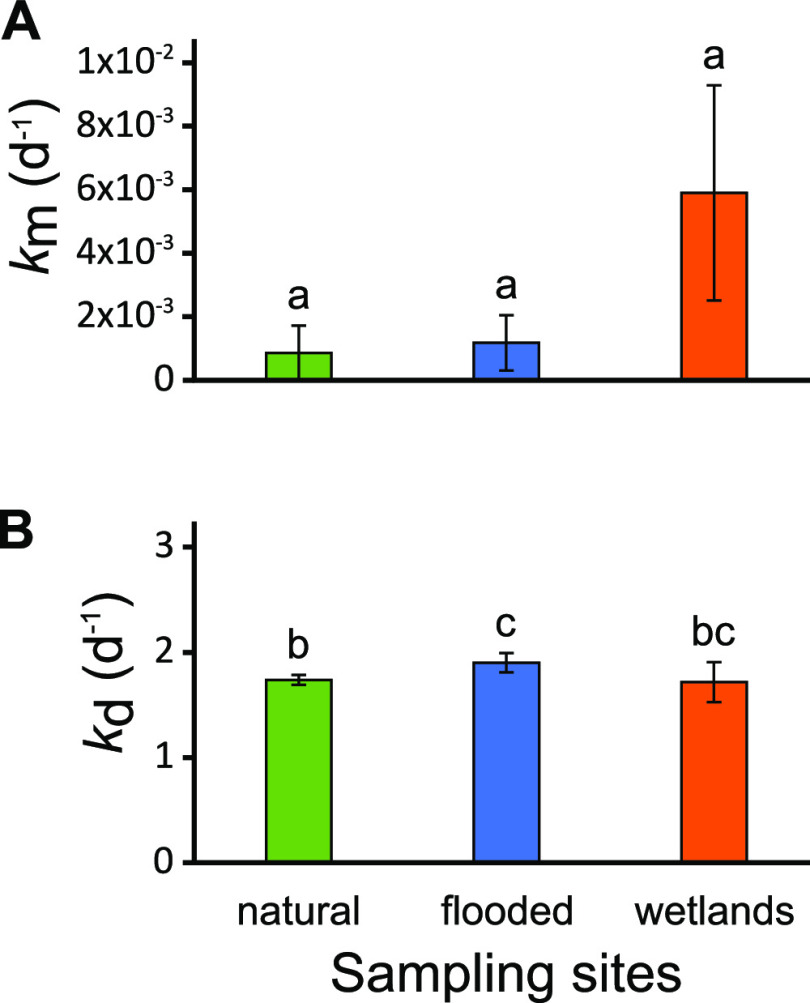
Periphyton methylation (A) and demethylation (B) rates for the studied sites. Bars are averaged values from triplicates, error bars are the standard error, and lowercase letters show significant differences between sites (*P* < 0.05).

Hg methylation and demethylation rates were measured under *in situ* conditions in periphyton at the natural, flooded, and constructed wetland sites (Fig. 2). Although variable among replicates, the highest mercury methylation rate (*k*_m_) was observed in the wetland periphyton samples, averaging 5.9 × 10^−3^ ± 3.4 × 10^−3^ day^−1^ (Fig. 2A). Methylation was detected in only a single replicate at each of the natural and flooded sites, where values were 2.6 × 10^−3^ day^−1^ and 2.9 × 10^−3^ day^−1^, respectively. However, differences in *k*_m_ between wetlands and the other two sites was not significant (*P* > 0.05), owing to high variability among replicates. In contrast to the limited distribution of detectable methylation rates, MMHg demethylation rates were similar for all three sites with a demethylation rate constant (*k*_d_) averaging 1.78 ± 0.21 day^−1^ (Fig. 2B). The natural and the flooded sites were significantly different from each other (*P* = 0.021), while the wetland site was not (*P* > 0.05). The *k*_m_ values in the constructed wetland periphyton were similar in magnitude to those previously reported from lake periphyton associated with green macroalgae (5) and about 100 times higher than those reported in an industrially impacted stream periphyton (28). For *k*_d_ values, averaged rates were about 10 times higher than those previously reported in the same two studies.

Recent studies suggested a transient availability rate potentials calculation, a modification of the original Hg methylation kinetic model from H. Hintelmann et al. (43) through the determination and addition of sorption reaction kinetics, leading to *k*_m_ 15 times higher than the full availability rate potentials (29, 44). However, the remoteness of our sites and field laboratory has prevented the determination of such additional sorption constants to fit the equations. Nevertheless, our experiment aimed to simulate natural conditions as accurately as possible by keeping periphyton disturbance at a minimum and performing incubations *in situ* at a depth corresponding to that of the sampled periphyton. Our study is among few to conduct Hg transformation assays in the field with untouched periphyton (i.e., not scraped from substrate and unmixed prior to incubation [6, 38]). Disruption of the periphytic matrix through homogenization of the biofilm has been shown to affect *k*_m_, leading to smaller yields (28). Stirring periphyton prior to incubation is likely to cause two main problems when estimating Hg methylation rates: (i) suboxic or anoxic microniches may be suppressed (45) and (ii) Hg availability may be enhanced through the disruption of natural diffusion kinetics into the biofilm (46), with unpredictable impacts on *k*_m_. *In situ* incubation was important to take in consideration for the representativeness of the experimental setup. Incubating periphyton at the depth from which the biofilm originated ensured an exposition to the natural light cycle and environmental temperature fluctuations. Several studies have shown the importance of light on methylation assays, suggesting an indirect implication of photosynthetic microorganisms in Hg methylation (16, 27, 28, 47), probably through the production of organic compounds enhancing methylation processes (42, 48). Water temperature is also a key factor controlling Hg methylation rates, where temperatures above 20°C have the highest yields in temperate zones (28, 47). Previous studies have shown a seasonal shift in periphyton net MMHg production where higher demethylation rates were measured in late summer (6). This is consistent with our results, where assays were conducted in August, and may partly explain the large difference in scale between *k*_m_ and *k*_d_ in wetlands.

### Bacterial and archaeal diversity in periphyton.

The differences in Hg transformation rates and relative concentrations of MMHg between the three sites provided an opportunity to identify microbial community composition, and the particular taxa, associated with Hg methylation in periphyton from different environments upstream of the Chute-Allard dam. To do so, we generated 16S rRNA gene (16S rDNA) and transcript (16S rRNA) data sets from nucleic acids extracted from the periphyton samples collected after the incubation periods (9 samples from each site: 3 for control and 6 for Hg transformation rates). The design allowed us to compare microbial community compositions between sites and simultaneously test for any effect of isotope addition and incubation on community composition (rDNA) and activity (rRNA).

**FIG 3:**
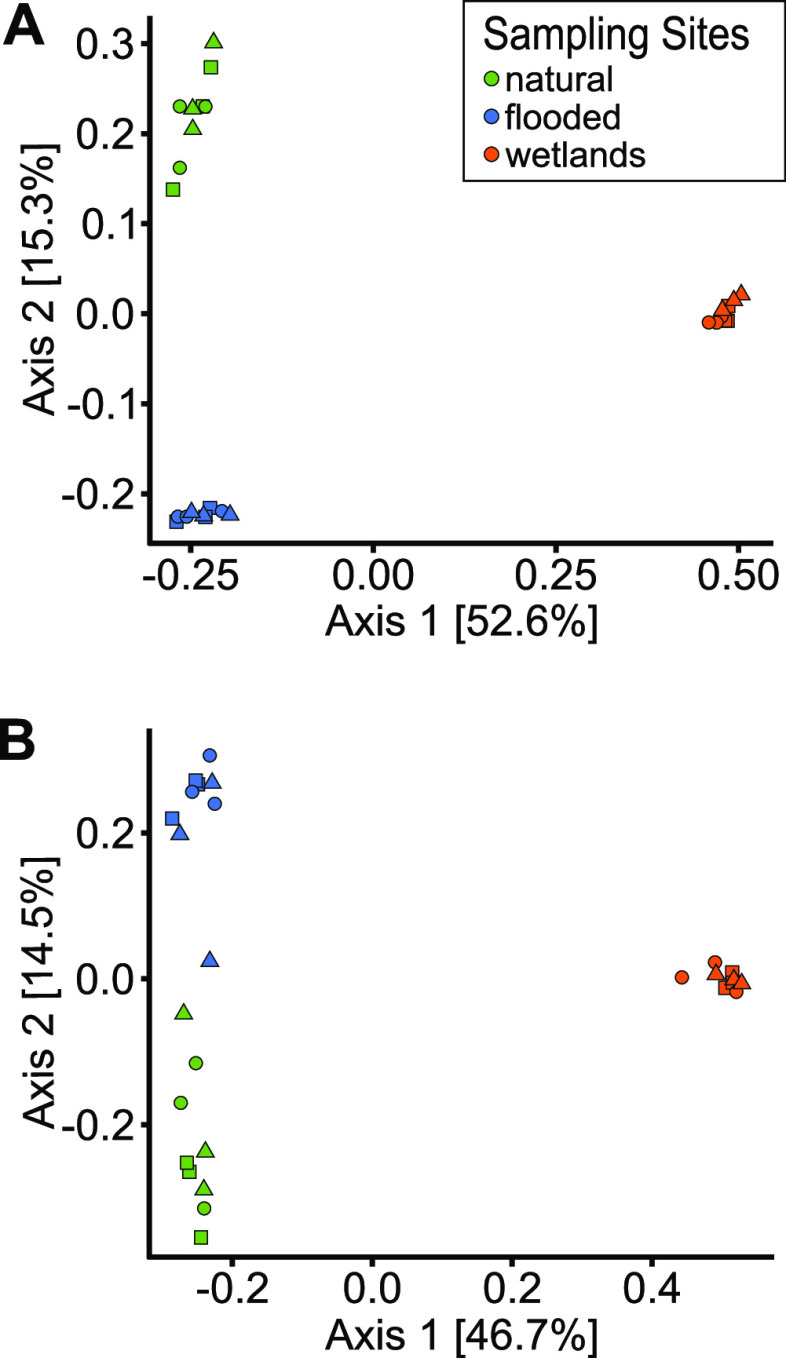
Principal-coordinate analysis based on Bray-Curtis dissimilarity matrices of 16S rDNA (A) and 16S rRNA (B) ASVs, where green, orange, and blue identify natural, flooded, and constructed wetland sites, respectively. Squares represent time zero, circles represent 48 h of incubation without Hg addition, and triangles represent 48 h of incubation with Hg addition.

Exploration of the 16S rDNA and rRNA data sets using principal-coordinate analysis (PCoA) ordination showed distinct community structures at the three sites. Constructed wetland samples were separated from natural and flooded sites along PC axis 1, which explained ∼50% of the variation in rDNA and rRNA data sets (Fig. 3). Sites were also separated along PC axis 2, although the variation explained (∼15%) was less than PC axis 1. In support of the ordination plot, Adonis analysis showed that samples were significantly grouped by sites in the rDNA and rRNA plots (*P* < 0.001), whereas the influence of experimental treatments was not significant (*P* = 0.2 for rDNA and *P* = 0.4 for rRNA) (Fig. 3).

In this study, the periphyton at the naturally submerged site originated from naturally occurring substrate, while those from the wetland and flooded sites were from artificial substrates. We observed slightly more variation among rDNA and rRNA data sets for the natural site, exemplified by less tightly clustered samples in the ordinations. Natural substrates such as rough decaying branches are likely to induce more heterogeneity within biofilms, and hence differences between sampling replicates, than the use of homogenous 1-year-old smooth and inert substrates (e.g., polypropylene mesh). Moreover, once established, thicker and older periphytic communities act as microcosms recycling internal nutrients (49) and are inclined to have multiple microniches within the biofilm and spatial-temporal (diurnal) changes in oxydo-reduction conditions (50). The use of artificial substrates may have an effect on periphyton diversity due to the colonization time, the biofilm thickness, the nature of the substrate, and the grazing pressure of invertebrates, for instance (51–53). However, as the artificial substrates were left for colonization for 1 year, their use for incubation should still be relevant for ecological interpretations for some natural substrates (e.g., macrophytes) as demonstrated by S. Hamelin (53). Nonetheless, it appears that the nature of the substrate was a minor factor influencing periphyton composition, compared to the larger qualitative differences in the environmental setting of the different periphyton locations.

### Taxonomic composition of periphyton communities.

**FIG 4:**
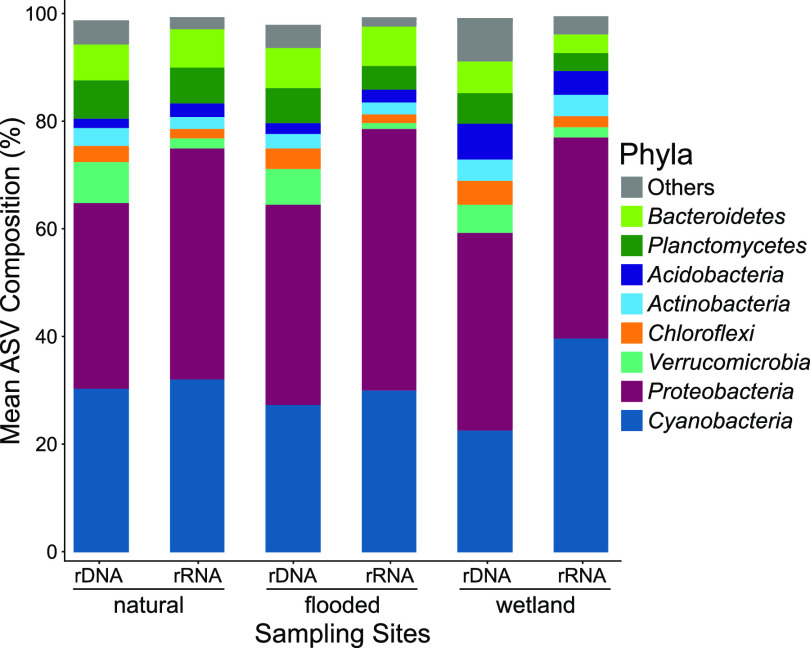
Bar plots of the mean relative abundance of periphyton bacterial phyla from 16S rDNA (genes) and rRNA (transcripts) ASVs for the three sampling sites. Phyla with relative abundance lower than 2% were combined as others; a comprehensive list can be found in Table S2.

A broad diversity of bacterial phyla was identified in periphyton at all sites. *Proteobacteria* was the most abundant phylum, averaging 36.3% ± 1.4% and 43.1% ± 5.6% of 16S rDNA and rRNA data sets, respectively. Second in abundance were *Cyanobacteria*, which averaged 26.8% ± 3.9% (rDNA) and 34.0% ± 5.1% (rRNA). Additional abundant phyla included *Verrucomicrobia*, *Acidobacteria*, and *Chloroflexi*. Overall, the wetland site exhibited the greatest observed phylum-level diversity for both 16S rDNA and rRNA data sets (Fig. 4). Complete bacterial phyla and their averaged relative abundance are listed in Table S2 in the supplemental material.

10.1128/mSphere.00021-21.4 TABLE S2 List of periphyton bacterial phyla and their averaged relative abundance (%) from 16S rDNA and rRNA amplicon sequence variants for the natural, flooded, and wetland sites. Phyla below the dashed line represent every bacterial phylum clustered as “others” in Fig. 4. Download Table S2, DOCX file, 0.03 MB. Copyright © 2021 Leclerc et al. 2021 Leclerc et al. https://creativecommons.org/licenses/by/4.0/ This content is distributed under the terms of the Creative Commons Attribution 4.0 International license.

**FIG 5:**
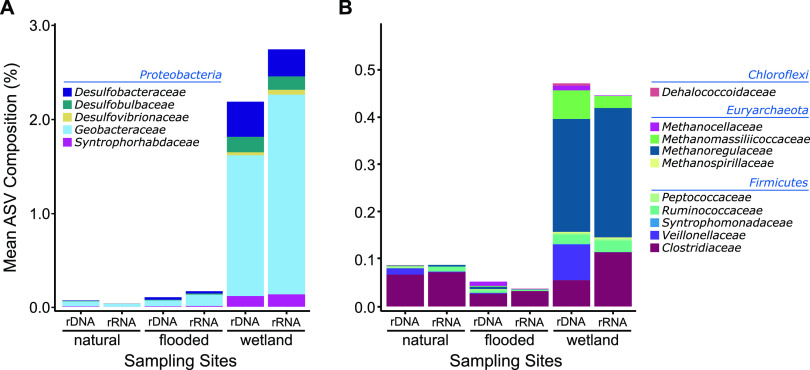
Bar plots of the mean read percent from 16S rDNA and rRNA ASVs of families associated with Hg-methylating main groups for *Proteobacteria* (A) and for *Euryarchaeota*, *Firmicutes*, and *Chloroflex*i (B). Phyla are in blue font, and families are in black font.

Given the elevated Hg methylation rates in the constructed wetland periphyton and the unique community structure identified by ordination analysis, we hypothesized that the wetland community was differentiated from the other sites by an abundance of anaerobic taxa with a capacity for MMHg production. Indeed, a comparison of communities at finer taxonomic resolution showed that Deltaproteobacteria families previously associated with Hg methylation were present at all sites but exhibited greatest relative abundance in the wetland site, averaging 2.6% and 2.2% for 16S rDNA and rRNA, respectively (Fig. 5A). The Deltaproteobacteria included families of SRB (*Desulfobacteraceae*, *Desulfobulbaceae*, and *Desulfovibrionaceae*), but it was the FeRB within *Geobacteraceae* that comprised the greatest proportion within wetland communities (2.1% of rDNA and 1.5% of rRNA). Methanogenic *Euryarchaeota* were also enriched at the wetland compared to natural and flooded sites (Fig. 5B). *Methanoregulaceae* were the most abundant methanogens in the wetland site, accounting for 0.23% of 16S rDNA and 0.27% of rRNA data sets. The observation of SRB, FeRB, and methanogens enriched within the wetland periphyton demonstrates that a diverse anaerobic community may be contributing the MMHg production in the wetlands of the St. Maurice River. Periphyton collected from the constructed wetlands was the thickest (>10 mm) of the three sites (insets of Fig. 1). Thickness can imply higher richness and diversity due to more microniches through the establishment of physicochemical gradients within the biofilms (50). These niches may allow the growth of aerobic microorganisms alongside sensitive phyla or families such as obligate anaerobes (54).

Putting these results alongside the methylation rate calculations (Fig. 2), a pattern can emerge where the periphyton with the highest relative abundance of known Hg-methylating families also has the highest rates of Hg methylation, although the small number of sites does not allow us to establish clear relationships. Conversely, G. A. Christensen et al. (55) concluded that community structure based on 16S amplicons was insufficient to obtain satisfactory relationships between putative methylating groups and Hg methylation. Even though MMHg ratio ([MMHg]/[THg]) may be used as an indicator for methylation capacity, which was the case in that study, it may not be representative enough of Hg-cycling dynamics to establish links between Hg methylation and the microbial community. In our study, no relationships were found between the putative Hg-methylating microorganisms based on 16S and the MMHg ratio. Periphyton from the wetland site had the highest relative abundance of known Hg-methylating families and was the one with the lowest MMHg ratio. It seems that methylation rates can be calculated to attempt linking the microbial community using 16S rDNA or rRNA data.

### Diversity of *hgcA* and *merAB* in periphyton metagenomes.

**FIG 6:**
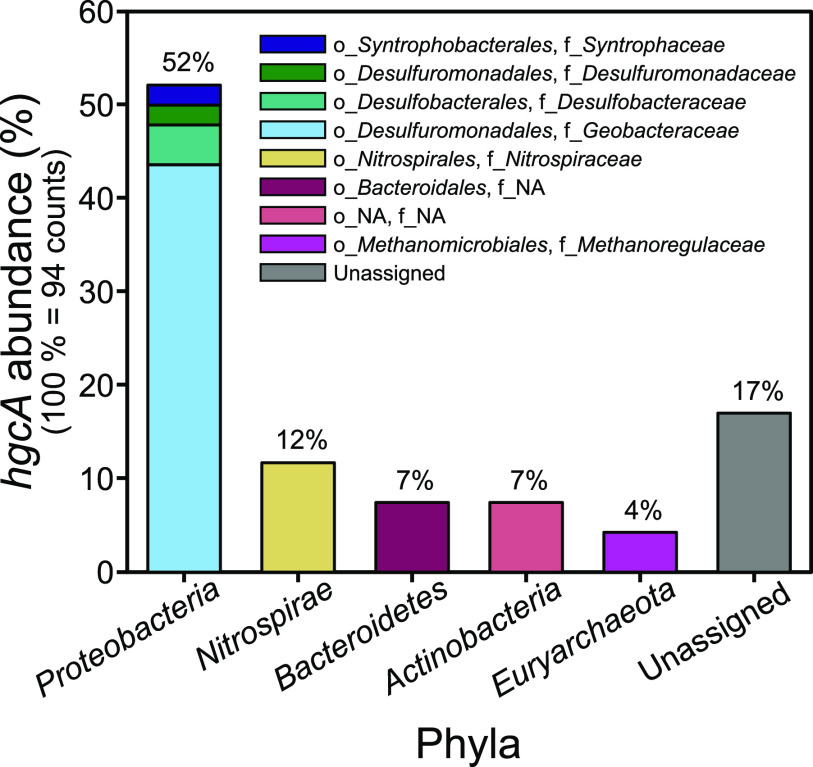
Abundance of the Hg-methylating *hgcA* gene from different phyla and their corresponding orders (o) and families (f) in periphyton sampled in the constructed wetland site.

The 16S rDNA and rRNA analysis demonstrated enrichment in known Hg-methylating taxa in constructed wetland periphyton communities compared to natural and flooded sites. However, given the patchy distribution of the genetic capacity for Hg methylation across genomes (23), it is challenging to assess methylation contributions from taxonomic composition alone. To further explore the Hg-methylating taxon diversity in the periphyton samples, 27 shotgun metagenomic data sets (9 from each of three sites) were generated and screened for the presence of *hgcA* genes (pooled by sites to increase their numbers). The *hgcA* gene was not detected in periphyton from the flooded or natural sites. However, a broad diversity of partial *hgcA* sequences was identified from the constructed wetland site. In total, 24 unique *hgcA* sequences were retrieved from the nine wetland metagenomes (see Table S1). Only partial *hgcA* sequences were detected (306 to 876 bp in length), and they were located on short scaffolds (308 to 1,372 bp in length), which precluded the possibility of reliable binning of metagenome-assembled genomes. Nevertheless, 20 partial *hgcA* sequences were taxonomically assigned to either *Proteobacteria* (52%), *Nitrospirae* (12%), *Actinobacteria* (7%), *Bacteroidete*s (7%), or methanogenic *Euryarchaeota* (4%) (Fig. 6), using a combined approach of blastp and phylogenetic tree construction for taxonomic assignment (Table S1 and Fig. S1). Eighty-three percent of putative HgcA sequences had the same taxonomy from blastp and the phylogenetic tree (including unassigned sequences) (Table S1 and Fig. S1). Concerning the four families of the phylum *Proteobacteria* (Fig. 6), we have especially high confidence in the taxonomic assignment of *Geobacteraceae*, since the reference HgcA sequences of this family formed a monophyletic clade, and our putative *Geobacteraceae* HgcA sequences were placed within this clade (Fig. S1). Reference *Syntrophaceae* HgcA sequences formed a distinct clade in the tree (with several exceptions) into which our putative *Syntrophaceae* HgcA sequence was placed, providing a satisfying level of confidence of our taxonomic assignment. However, our putative *Desulfobacteraceae* and *Desulfuromonadaceae* HgcA sequences received their taxonomy assignments from the blastp approach but were unassigned from the tree output. Concerning taxa of additional phyla (Fig. 6), our *Nitrospiraceae* HgcA sequences achieved high taxonomic confidence, since all of them were placed into a distinctly clustered *Nitrospiraceae* clade in the phylogeny (Fig. S1). The same pattern was observed for our *Methanomicrobiales* HgcA sequences. Concerning *Actinobacteria*, the reference HgcA sequences belonging to this phylum were located throughout the tree, but one distinct clade contained only *Actinobacteria*, into which our putative *Actinobacteria* HgcA sequences were placed, which further supports their assigned blastp taxonomy. In contrast, our putative *Bacteroidales* HgcA sequences (taxonomy from blastp output) were unassigned from the tree, since they were not placed into any distinct clade. To conclude, our *Desulfuromonadaceae*, *Desulfobacteraceae*, and *Bacteroidales* sequences represent the 17% of our taxa with a rather hypothetical taxonomic assignment, while taxonomic assignments of the remaining 83% seemed to be consistent between sequencing similarity and phylogenetic location with reference HgcA sequences.

10.1128/mSphere.00021-21.1 FIG S1 Putative HgcA genes from the wetlands (turquoise, contig number followed by taxonomy) embedded within HgcA database (black). Collapsed clades (triangles) represent various taxa. Download FIG S1, EPS file, 2.0 MB. Copyright © 2021 Leclerc et al. 2021 Leclerc et al. https://creativecommons.org/licenses/by/4.0/ This content is distributed under the terms of the Creative Commons Attribution 4.0 International license.

10.1128/mSphere.00021-21.3 TABLE S1 List of putative HgcA sequences from the wetlands with taxonomies from blastp and phylogenetic tree, contig characteristics, and hmm scores. The sequences are color coded according to the level of confidence for identification as HgcA and taxonomic assignment. Download Table S1, XLSX file, 0.02 MB. Copyright © 2021 Leclerc et al. 2021 Leclerc et al. https://creativecommons.org/licenses/by/4.0/ This content is distributed under the terms of the Creative Commons Attribution 4.0 International license.

Finer taxonomic resolution showed that *Geobacteraceae* were the dominant contributors to *hgcA* abundance in wetland periphyton communities, along with families of SRB and *Methanoregulaceae* (Fig. 6). Other studies have identified SRB and methanogen *hgcA* sequences in periphyton but not the dominance of *Geobacteraceae*. S. Bouchet et al. (5) reported the *Desulfobulbus* genus, an SRB, to be the dominant putative Hg methylator in periphyton associated with Characeae (a class of charophyte, a green macroalga) with a relative abundance reaching 36%. *Geobacter* was also identified but at a relative abundance about six times less than *Desulfobulbus*. Sulfate reducers were found to be dominant putative Hg methylators in periphyton associated with the roots of the water plant *Ludwigia* (12). Interestingly, periphyton from the Everglades had a different profile, with syntrophs associated with high concentrations of sulfate and methanogens as the most abundant putative Hg methylators (26). *In situ* experiments using specific inhibitors previously identified Hg methylation to be dominated by sulfate reducers or methanogens in periphyton (14, 16, 38, 47). However, the combination of 16S rRNA analysis and the *hgcA* genes recovered from metagenomes suggests that FeRB are key players in Hg methylation in periphyton from the wetland site.

Next, we assessed diversity of Hg-demethylating microbes in metagenomes. The organomercury lyase gene (*merB*) responsible for demethylation was not detected in any metagenomes, although a few mercuric reductase (*merA*) genes were present (Fig. S2). Given that the *mer* operon is typically absent in anaerobic organisms (56), these results suggest that processes other than aerobic reductive demethylation are responsible for the demethylation observed in the periphyton communities. The anaerobic oxidative demethylation mechanisms are still unclear, but they have been suggested to be related to the oxidation of acetate by SRB or to the methylamine degradation by methanogens (31). The *in situ* experiments having been carried out in the photic zone, it is likely that the observed MMHg demethylation was mainly abiotic.

10.1128/mSphere.00021-21.2 FIG S2 Bar plot of the number of *merA* gene markers found at each site for every replicate. Download FIG S2, EPS file, 0.5 MB. Copyright © 2021 Leclerc et al. 2021 Leclerc et al. https://creativecommons.org/licenses/by/4.0/ This content is distributed under the terms of the Creative Commons Attribution 4.0 International license.

### Conclusions.

The combined use of *in situ* rate measurements and assessment of the genetic determinants for Hg methylation in periphyton demonstrated a relatively high capacity for MMHg production in constructed wetlands compared to the flooded and natural sites upstream of the St. Maurice River run-of-river hydroelectric dam. The wetland site exhibited the thickest biofilms, favoring Hg-methylating niches such as suboxic environments or gradients of redox conditions. This is consistent with the screening results for the *hgcA* gene, where periphyton from the wetlands site was the only one where *hgcA* could be identified (Fig. 5). Since *hgcA* was challenging to find in samples, we do not exclude the possibility that some periphytic microorganisms from the flooded and natural sites possessed the Hg-methylation gene cluster, accounting for the low *k*_m_ measured in some of the replicates. However, as shown by previous studies conducted in laboratory with pure cultures (18) or in aquatic environments with natural matrices (5), the presence of the *hgcAB* gene cluster appears to be the first requirement for methylation of Hg. MMHg concentrations have been found to poorly correlate with *hgcAB* abundance (55). In our study, we identify Deltaproteobacteria, especially from the putative FeRB *Geobacteraceae*, as likely key contributors in periphytic Hg methylation.

Once biologically produced, MMHg is often rapidly exported out of the cell (57). However, in the case of periphyton, whether this newly exported MMHg remains trapped in the periphytic matrix, adsorbed to cell surfaces or any extracellular ligands such as various exopolymeric substances constituting the biofilm structure (48), or is transferred out of the periphyton is unknown. Given the high MMHg concentration detected in the constructed wetland water compared to the MMHg concentration found in the biofilms (Table 1), it is likely that periphyton plays some role as a source of MMHg for the surrounding water of this site rather than a sink. Moreover, a parallel study conducted in the same river area did not find a high abundance of *hgcA* nor a high concentration of MMHg in the sediments (0.31 ± 0.20 ng g^−1^ of DW, representing 1.59% ± 0.58% of THg) of the constructed wetland site compared to the flooded and natural sites (58). This does not detract from the likely implications of sediments in the production and export of MMHg in the wetland site. However, periphyton can methylate Hg with higher rates than sediments, as shown by S. Hamelin et al. (6), where net MMHg production of periphyton was estimated to be 2 orders of magnitude higher than within sediments from Lake St. Pierre (an enlargement of the St. Lawrence River, Québec, Canada). With this knowledge, although MMHg fluxes were not measured between periphytic biofilms, sediments, and the surrounding water, it seems that periphyton from the constructed wetland site may act as an important source of MMHg, especially considering the expansive periphyton coverage at this site. Further investigations are needed, however, including estimation of *k*_m_ from sediments, to properly compare periphyton and sediments along the Chute-Allard pondage of the St. Maurice River.

Overall, the results of the present work suggest that the construction of artificial wetlands has the potential to increase MMHg production through the creation of habitats promoting periphyton growth and thus anaerobic assemblages associated with Hg methylation. However, as the artificial wetlands were also highly productive sites, heavily colonized by macrophytes and algae, and with abundant invertebrates and small fish, it is challenging to extrapolate effects of periphyton methylation capabilities on local wildlife MMHg levels. It is unclear at this point to establish whether the construction of the run-of-river dam may have been responsible for an increase in MMHg production by periphyton. The chosen flooded site was only one of many heterogeneous habitats found in the pondage area (including several scattered bays). River impoundments create habitats from riverine to lacustrine zones (59). However, our sampling sites were located in either the mixed riverine or transition zone with no sites in the main water storage area. Among possible future research, a more extensive coverage of the periphytic habitats created by the flooding such as wetlands, swamps, and the reservoir lacustrine-like zone (covering a small area for run-of-river dams) is advised. Moreover, understanding the behavior of MMHg from periphytic origins through bioaccumulation and biomagnification processes along benthic trophic chains should be the next step for the management of such artificial habitats.

## MATERIALS AND METHODS

### Site description.

The three studied sites were located upstream of the run-of-river hydroelectric power plant Chute-Allard on the St. Maurice River in the Precambrian Shield in Québec, Canada (47°53′34″ N, 73°43′5″ W) (Fig. 1). The constructed wetland site was located upstream of the pondage in artificial channels designed for wildlife conservation and spawning of yellow perch through the creation of wetland areas. The flooded and natural sites were both located in the pondage of the hydroelectric dam, where the flooded site was in a newly submerged area and the natural site was part of the original river flow. For wetland and flooded sites, artificial substrates made of polypropylene mesh fixed on an acrylic frame (28) were installed at a depth of 1 m in 2016 to create uniform colonization sites for periphyton and were collected after 1 year, in late summer of 2017.

### Sampling.

The day before incubation, site-specific water was collected with a peristaltic pump connected to a groundwater filtering system (pore size of 0.45 μm; Pall) for dissolved organic carbon (DOC), MMHg, and total Hg (THg) analyses. Norprene and Teflon tubing were cleaned with a 10% HCl solution and subsequently rinsed with Milli-Q (18.2 MΩ · cm) and river water before sampling each site. For Hg transformation incubation experiments, water was further filtered at 0.2 μm (polycarbonate filters; Millipore) using a filtration tower back in the laboratory. Water was either spiked with stable isotope-enriched solutions (MM^198^Hg and ^200^Hg; National Research Council, Canada, and Trace Sciences International, USA, respectively) for Hg transformation rate calculation or kept unaltered for genetic controls. When amended, final concentrations of Hg were ∼4 ng liter^−1^ for each enriched isotope, which represent about 25 and 3 times the natural water concentrations of MMHg and THg, respectively. Spiked water was left to equilibrate overnight before the experiment. On the day of the incubation, the artificial substrates were pulled out of the water and then subsampled to obtain periphyton replicates of 4 cm^2^. As natural substrates, 10-cm-long wood branches colonized by periphytic biofilms were sampled from a submerged tree for the natural site samples using 0.68-liter Pac-man boxes, a modified version (by C. Vis, Parks Canada) of the 6-liter Downing box (60). Periphyton was manipulated with great care to avoid contamination and structural disturbance and to minimize loss of material. Water dissolved oxygen, redox potential, and pH values were recorded using a YSI Pro Plus multiparameter probe (Xylem, Yellow Springs, OH, USA). Sample collection and manipulation on the field were performed under the “clean hands, dirty hands” protocol appropriate for trace metal sampling (61). Glassware was acid washed (5% HCl; 45% HNO_3_) overnight as well as plasticware (10% HCl), rinsed three times with Milli-Q water in the laboratory, and then rinsed three times with 0.45-μm-filtered river water prior to sampling.

### Incubation experiments.

Artificial substrates colonized by periphyton were fixed to a high-density polyethylene tube prior to incubation, and tree branches were directly fixed inside polycarbonate incubation bottles previously filled with 150 ml of site-specific equilibrated filtered water (unamended or spiked with isotope-enriched solutions as described above). Incubation bottles were randomly distributed horizontally along a tubular support submerged at the location and depth from where periphyton originated to maintain as much as possible the same environmental conditions. Treatments were performed in triplicates for a total of 9 samples per site (Hg transformation rates at *t*_0_ and *t*_48h_ and a control incubated 48 h without Hg amendment). After *in situ* incubation, samples for transformation rate analyses were acidified with 900 μl of 4M HCl (Omnitrace Ultra; MilliporeSigma) to avoid further Hg transformations. In the laboratory, periphyton was brushed from substrates with an acid-cleaned toothbrush and then stirred until homogenization. Between 15 and 25 ml of suspended material was filtered on precombusted GF/F filters for dry weight (DW) and ash-free dry weight (AFDW) measurements. For transformation rate analyses, samples were stored about 3 weeks at −20°C and then freeze-dried for storage until analyses (Freeze-Dry System; Labconco, Kansas City, MO, USA). For genomic analyses, between 15 and 25 ml of the suspended solution was filtered through a 0.45-μm polycarbonate filter and the filtrate went through a 0.2-μm polycarbonate filter (Millipore). Filters were put together in a cryovial, flash-frozen in liquid nitrogen, and kept at −80°C until extractions.

### DOC, DW, and AFDW measurements.

DOC in water was measured as nonpurgeable organic carbon with an Aurora 1030 total organic carbon (TOC) analyzer (OI Analytical, College Station, TX, USA) after the addition of H_3_PO_4_ and digestion with persulfate. Precombusted GF/F filters with periphyton were dried for 48 h at 65°C and then combusted for 2 h at 550°C for DW and AFDW measurements, respectively.

### Mercury analyses.

For MMHg analyses, water samples were distilled prior to analysis using 45 ml of diluted samples under nitrogen flow at 130°C. The distillate was supplemented with 40 μl of 2.5% (wt/vol) ascorbic acid and incubated for 15 min. Freeze-dried periphyton was weighted with a microbalance prior to a first digestion overnight at 65°C using 500 μl of 33% (vol/vol) HNO_3_. Protocols for MMHg measurements followed U.S. EPA method 1630 using cold vapor atomic fluorescence spectrophotometry (CVAFS; Tekran 2700; Tekran Instruments Corporation, Toronto, Canada). Ethylation was performed using sodium tetraethylborate (NaBEt_4_) after the addition of acetate buffer. For THg analyses, the leftover digesta went through a second digestion adding 583 μl of HCl-HNO_3_ solution (64.3% HCl and 35.7% HNO_3_, vol/vol; Omnitrace Ultra; MilliporeSigma) and was autoclaved with an electric sterilizer for 3 h (All American, Manitowoc, WI, USA; 121°C, 15 lb/in^2^) followed by the addition of 250 μl of H_2_O_2_ (9.79 M, Optima grade). The new digesta was then left overnight at room temperature. THg measurements in water and periphyton were made following U.S. EPA method 1631 using CVAFS (Tekran 2700; Tekran Instruments Corporation, Toronto, Canada). Oxidization of samples occurred with BrCl before reduction with SnCl_2_ and then preconcentration on a gold amalgamator. Analytical stability controls were performed running 0.5 ng liter^−1^ and 2.0 ng liter^−1^ of new standards after each set of 10 samples for MMHg and THg, respectively. Certified reference material from National Research Council of Canada TORT-2 was used for MMHg quality control, and both MMHg and THg analyses met the Canadian Association for Laboratory Accreditation (CALA) intercalibration criteria. The detection limits were 0.01 ng liter^−1^ for MMHg and 0.04 ng liter^−1^ for THg. Hg isotopes were separated through CVAFS before injection in inductively coupled plasma tandem mass spectrometry (Agilent 8900 triple quadrupole ICP-MS, Santa Clara, CA, USA). IHg concentrations were estimated by subtraction of MMHg from THg concentrations.

### Mercury transformation rate constant calculation.

Calculation of methylation and demethylation rate constants was performed using a first-order kinetics model for net methylmercury production (43) written as follows:

(1) $$\frac{\text{d}[\text{MMHg}]}{\text{d}t}=k_{\text{m}}[{\text{Hg}}^{\text{II}}]\;-\;k_{\text{d}}[\text{MMHg}]$$

where [MMHg] is the concentration of MMHg, [Hg^II^] is the concentration of IHg, *k*_m_ is methylation rate constant (in units of day^−1^), and *k*_d_ is demethylation rate constant (in units of day^−1^). With spiked isotope tracer assay, initial [MM^200^Hg] and [^198^Hg^II^] are assumed to be zero, which allows a simplification of equation 1, resulting in the following equations for Hg transformation rate constants after integration:

(2) km= −ln(1 − [MM200Hg]t[H200gII]0)t

(3) $$k_{\text{d}}=-\text{ln}\left(\frac{{\left[{\text{MM}}^{198}\text{Hg}\right]}_{t}}{{\left[{\text{MM}}^{198}\text{Hg}\right]}_{0}}\right)/t$$

where [^200^Hg^II^]_0_ and [MM^198^Hg]_0_ are initial added tracer concentrations in incubation bottles; [MM^200^Hg]*_t_* and [MM^198^Hg]*_t_* are tracer concentrations at time *t*, and *t* is incubation time. As experiments yielded a single time point measurement, in addition to an initial spiked concentration (*t*_0_), tracer concentrations were directly fitted into equations 2 and 3 (28). Triplicates were used for transformation rate means and standard error estimations. Analyses of variance were performed using the *aov* function with R software (62) to compare Hg transformation rates between sites.

### Nucleic acid extractions.

DNA was extracted from periphyton using the DNeasy PowerWater kit (Qiagen) following the standard protocol and with the following modifications: (i) a 10-min incubation at 65°C was included after the addition of solution PW1 for cell lysis and (ii) the cell lysate was treated with 1 μl of RNase (Thermo Fisher Scientific) followed by incubation at 37°C for 30 min. RNA was extracted from periphyton using the RNeasy PowerWater kit (Qiagen) protocol with the following modification: a 10-min incubation at 65°C was added after the addition of solution PM1/beta-mercaptoethanol (β-ME) for cell lysis.

### 16S rRNA gene PCR, RT-PCR, and amplicon sequencing.

For 16S rRNA gene analysis, PCR amplification of the V4 region of the 16S rRNA genes was performed with primers 515FB (5′ GTGYCAGCMGCCGCGGTAA 3′) (63) and 806RB (5′ GGACTACNVGGGTWTCTAAT 3′) (64) containing CS1 (5′-ACACTGACGACATGGTTCTACA-3′) and CS2 (5′-TACGGTAGCAGAGACTTGGTCT-3′) adapters for Illumina sequencing. The 25-μl PCR mixture consisted of 5 μl of 5× Phusion Reaction HF buffer, 0.5 μl deoxynucleoside triphosphates (dNTPs) (10 mM), 0.5 μl Phusion HF DNA polymerase (2,000 U ml^−1^), 1.25 μl of the forward and reverse primers, and 1 μl of DNA. PCR cycling conditions were as follows: 98°C for 1 min and cycling 35 times at 98°C for 10 s, 55°C for 30 s, and 72°C for 20 s, with a final incubation at 72°C for 5 min.

For 16S rRNA transcript analysis, reverse transcription was performed using the TaqMan reverse transcription reagent kit (Applied Biosystems). A 10-μl reaction mixture was used for each sample consisting of 1.0 μl 10× TaqMan RT buffer, 2.2 μl 25 mM magnesium chloride, 2.0 μl dNTP mixture, 0.5 μl 806RB primer, 0.2 μl RNase inhibitor, 0.25 μl MultiScribe reverse transcriptase (50 U μl^−1^), and 3.0 μl RNA. The reaction mixtures were incubated at 48°C for 30 min, and then temperature was increased to 95°C for 5 min to inactivate the reverse transcriptase. The cDNA was used as the template for PCR using the same protocol as described above. Multiplex amplicon sequencing was performed using Illumina MiSeq and 250-bp paired-end technology at the Genome Quebec Innovation Centre at McGill University.

### 16S rRNA amplicon analysis.

Sample demultiplexing, read quality control (QC), merging of paired-end reads, and generation of amplicon sequence variants (ASVs) and taxonomic assignments were performed with the DADA2 Pipeline 1.6 (65) with the following details. Quality profiles were created for each forward and reverse fastq sequence and then trimmed at 220 and 180 for the forward and reverse reads, respectively. The first 19 bp and 20 bp were removed from the forward and reverse reads to remove the 515BAF and 806BAR primers, respectively. The forward and reverse reads were then dereplicated to combine all identical sequences into “unique sequences” with the corresponding abundance. Real sequence variants were identified by applying the core sequence-variant inference algorithm, and the paired forward and reverse reads were merged. A sequence table was then constructed, and the chimeras were removed. Taxonomy was assigned down to the species level using the silva_nr_v132_train_set.fa.gz and silva_species_assignment_v132.fa.gz (66). The *phyloseq* R-package (67) was used to analyze and visualize diversity and taxonomic composition of samples. Principal-coordinate analysis (PCoA) was performed using relative ASV abundances and Bray-Curtis distance measure. A permutational (999 permutations) multivariate analysis of variance (MANOVA) using distance matrices was completed in conjunction with the PCoA using the Adonis test from the *vegan* R-package (68) for significance.

### Metagenome sequencing, assembly, and annotation.

Shotgun sequencing was performed using Illumina NovaSeq 6000 S4 PE150 technology at the Genome Quebec Innovation Centre at McGill University. Reads were quality trimmed and filtered using Trimmomatic v.0.38 (69), and metagenome assemblies were generated by Megahit v1.0.6 (–k-list 23, 43, 63, 83, 103, 123) (70). The Burrows-Wheeler Alignment Maximum Exact Matches (BWA-MEM) tool was used with option -bwtsw (71) to index the scaffolds and to perform alignment of paired-end reads. The scaffolds and average scaffold depth of coverage files (created with the jgi_summarize_bam_contig_depth script [72]) were submitted to the Joint Genome Institute Integrated Microbial Genomes and Microbiomes (JGI IMG/M) platform for gene identification and functional annotation.

### HgcA and MerAB identification, abundance, and taxonomy.

The annotated protein sequences produced by the JGI IMG/M pipeline were screened for sequences containing HgcA by an hmm search (73) against an HgcA protein sequence hmm model (25). Hits with a score of ≥100 were considered HgcA sequences and were multiplied with their coverage to calculate their abundance. The score was set based on alignments against HgcA sequences from the reference database and identification of the more conserved motifs (NVWCAAGK, NVWCASGK, NVWCAGGK, NIWCAAGK, NIWCAGGK, or NVWCSAGK). Putative HgcA sequences with a score of ≥164 contained the motif. Putative HgcA sequences with a score of ≥100 still aligned well with the HgcA database but missed the motif region, since the contigs were too short to contain the complete *hgcA* gene. The level of confidence for each putative HgcA sequence can be found in Table S1 in the supplemental material. As there were only a few hits, outputs of all samples were grouped by sites (natural, flooded, and wetlands) for a comprehensive overview. The HgcA sequences were taxonomically assigned with blastp in diamond, version 0.9.30 (74), to an HgcAB sequence database containing 650 sequences (25). Threshold values (–E value 1E−20, –id 40, –query-cover 80) were chosen by comparison between the blast results (Table S1) and a phylogenetic approach to assign taxonomy to the HgcA sequences (Fig. S1). For the latter, HgcA sequences from our study and 650 sequences from the HgcAB database (25) were aligned using MUSCLE in MEGA6 (75). Aligned positions with weights of <0.5 were masked using the probabilistic masker ZORRO39 (76). The concatenated alignment consisted of 313 amino acid positions. Phylogenetic tree construction was done with MEGA6 based on maximum likelihood using 100 replicates, a JTT substitution model, a gamma distribution with invariant sites model for the rate variation with four discrete gamma categories, and the nearest-neighbor interchange (NNI) heuristic search method. Functional gene markers coding for MerA, a mercuric reductase responsible for the reduction of Hg^II+^ to Hg^0^, and MerB, an organomercury lyase that cleaves carbon-Hg bonds (31), were searched based on their Enzyme Commission (EC) numbers (MerA = 1.16.1.1, MerB = 4.99.1.2) in the annotations provided by JGI IMG/M.

### Data availability.

All 16S rRNA amplicon data are available at NCBI SRA under BioProject PRJNA702110. All assembled metagenomic data are available at IMG/M under GOLD study ID Gs0140997. Putative partial HgcA sequences are listed in Table S1.
