# Supplementary material for: Microbial Diversity and Mercury Methylation Activity in Periphytic Biofilms at a Run-of-River Hydroelectric Dam and Constructed Wetlands
Source: mSphere. 2021 Mar 17;6(2):e00021-21. doi: 10.1128/mSphere.00021-21 (PMC8546676; doi:10.1128/mSphere.00021-21)
Supplement: TABLE S2 [file msphere.00021-21-st002.docx]

## **Table S2.** List of periphyton bacterial phyla and their averaged relative abundance (%) from 16S rDNA and rRNA amplicon sequence variants for the natural, flooded, and wetland sites. Phyla below the dashed line represent every bacterial phylum clustered as “others” in **Fig. 4**.

|  | **Natural** | | **Flooded** | | **Wetland** | |
| --- | --- | --- | --- | --- | --- | --- |
| **Phyla** | rDNA | rRNA | rDNA | rRNA | rDNA | rRNA |
| Proteobacteria | 34.5879 | 42.9910 | 37.4159 | 48.8258 | 36.7912 | 37.4672 |
| Cyanobacteria | 30.3975 | 32.2132 | 27.3258 | 30.0569 | 22.6319 | 39.7532 |
| Bacteroidetes | 6.5522 | 7.2362 | 7.5063 | 7.3677 | 5.9284 | 3.3926 |
| Planctomycetes | 7.1921 | 6.6850 | 6.4766 | 4.5029 | 5.6553 | 3.3525 |
| Verrucomicrobia | 7.6920 | 1.8164 | 6.5616 | 1.1029 | 5.2114 | 1.9393 |
| Actinobacteria | 3.3729 | 2.1521 | 2.8186 | 2.2083 | 3.9211 | 4.0247 |
| Acidobacteria | 1.6974 | 2.5109 | 2.0387 | 2.3674 | 6.7485 | 4.4133 |
| Chloroflexi | 3.0015 | 1.8606 | 3.8372 | 1.5326 | 4.5489 | 2.0465 |
| Armatimonadetes | 0.9431 | 0.6903 | 1.3840 | 0.6273 | 0.5575 | 0.1734 |
| Patescibacteria | 1.0886 | 0.0431 | 0.8556 | 0.0166 | 1.6211 | 0.0219 |
| Spirochaetes | 0.6217 | 0.2786 | 0.5068 | 0.1750 | 0.7368 | 0.6076 |
| Nitrospirae | 0.1469 | 0.0806 | 0.1077 | 0.0436 | 1.1611 | 0.5669 |
| Gemmatimonadetes | 0.5505 | 0.2526 | 0.4121 | 0.1891 | 0.4687 | 0.2775 |
| Euryarchaeota | 0.0208 | 0.0091 | 0.0786 | 0.0151 | 0.7824 | 0.6607 |
| Chlamydiae | 0.2312 | 0.2491 | 0.0702 | 0.0899 | 0.2388 | 0.0947 |
| Latescibacteria | 0.1858 | 0.0343 | 0.2395 | 0.0378 | 0.3872 | 0.0262 |
| Firmicutes | 0.1160 | 0.1195 | 0.0612 | 0.0430 | 0.2294 | 0.2230 |
| Crenarchaeota | 0.0017 | 0.0000 | 0.0294 | 0.0008 | 0.3105 | 0.0191 |
| Rokubacteria | 0.0052 | 0.0021 | 0.0161 | 0.0013 | 0.2418 | 0.0725 |
| Dependentiae | 0.0692 | 0.0652 | 0.0545 | 0.0413 | 0.0599 | 0.0359 |
| Kiritimatiellaeota | 0.0056 | 0.0070 | 0.0389 | 0.0295 | 0.1506 | 0.0853 |
| BRC1 | 0.0621 | 0.0298 | 0.0740 | 0.0227 | 0.0300 | 0.0021 |
| Omnitrophicaeota | 0.0046 | 0.0242 | 0.0124 | 0.0239 | 0.0968 | 0.0588 |
| WPS-2 | 0.0448 | 0.0081 | 0.0649 | 0.0136 | 0.0700 | 0.0171 |
| Elusimicrobia | 0.0137 | 0.0056 | 0.0340 | 0.0068 | 0.1193 | 0.0285 |
| Nanoarchaeaeota | 0.0055 | 0.0060 | 0.0144 | 0.0093 | 0.0890 | 0.0490 |
| Fibrobacteres | 0.0122 | 0.0084 | 0.0122 | 0.0076 | 0.0470 | 0.0697 |
| FCPU426 | 0.0400 | 0.0424 | 0.0052 | 0.0015 | 0.0446 | 0.0076 |
| Zixibacteria | 0.0286 | 0.0025 | 0.0041 | 0.0025 | 0.0611 | 0.0207 |
| Hydrogenedentes | 0.0189 | 0.0021 | 0.0164 | 0.0088 | 0.0624 | 0.0083 |
| Thaumarchaeota | 0.0256 | 0.0000 | 0.0000 | 0.0000 | 0.0641 | 0.0064 |
| Deinococcus-Thermus | 0.0298 | 0.0011 | 0.0265 | 0.0000 | 0.0018 | 0.0000 |
| Lentisphaerae | 0.0011 | 0.0000 | 0.0006 | 0.0005 | 0.0271 | 0.0176 |
| Nitrospinae | 0.0020 | 0.0000 | 0.0006 | 0.0000 | 0.0350 | 0.0010 |
| Fusobacteria | 0.0005 | 0.0000 | 0.0048 | 0.0000 | 0.0119 | 0.0096 |
| Modulibacteria | 0.0005 | 0.0018 | 0.0000 | 0.0000 | 0.0139 | 0.0045 |

*Continuation of Table S1.*

|  | **Natural** | | **Flooded** | | **Wetland** | |
| --- | --- | --- | --- | --- | --- | --- |
| **Phyla** | rDNA | rRNA | rDNA | rRNA | rDNA | rRNA |
| Epsilonbacteraeota | 0.0048 | 0.0147 | 0.0000 | 0.0020 | 0.0009 | 0.0010 |
| Cloacimonetes | 0.0000 | 0.0000 | 0.0004 | 0.0000 | 0.0183 | 0.0023 |
| WS4 | 0.0000 | 0.0000 | 0.0013 | 0.0000 | 0.0160 | 0.0000 |
| Diapherotrites | 0.0000 | 0.0000 | 0.0000 | 0.0000 | 0.0084 | 0.0000 |
| Firestonebacteria | 0.0000 | 0.0000 | 0.0000 | 0.0000 | 0.0017 | 0.0035 |
| Margulisbacteria | 0.0058 | 0.0025 | 0.0075 | 0.0055 | 0.0020 | 0.0010 |
| WS1 | 0.0003 | 0.0000 | 0.0000 | 0.0000 | 0.0149 | 0.0012 |
| Mollusca | 0.0000 | 0.0000 | 0.0000 | 0.0000 | 0.0000 | 0.0027 |
| Annelida | 0.0000 | 0.0102 | 0.0000 | 0.0000 | 0.0000 | 0.0024 |
| Caldiserica | 0.0000 | 0.0000 | 0.0000 | 0.0000 | 0.0035 | 0.0012 |
| Tardigrada | 0.0000 | 0.0011 | 0.0000 | 0.0000 | 0.0000 | 0.0036 |
| Tenericutes | 0.0016 | 0.0000 | 0.0024 | 0.0008 | 0.0018 | 0.0004 |
| WOR-1 | 0.0000 | 0.0000 | 0.0000 | 0.0000 | 0.0051 | 0.0000 |
| Altiarchaeota | 0.0000 | 0.0000 | 0.0000 | 0.0000 | 0.0021 | 0.0023 |
| WS2 | 0.0000 | 0.0000 | 0.0004 | 0.0000 | 0.0057 | 0.0000 |
| Ochrophyta | 0.0000 | 0.0035 | 0.0000 | 0.0113 | 0.0000 | 0.0065 |
| Acetothermia | 0.0005 | 0.0000 | 0.0000 | 0.0000 | 0.0024 | 0.0000 |
| Poribacteria | 0.0000 | 0.0000 | 0.0000 | 0.0000 | 0.0013 | 0.0000 |
| FBP | 0.0009 | 0.0007 | 0.0004 | 0.0005 | 0.0009 | 0.0000 |
| Rotifera | 0.0000 | 0.0000 | 0.0000 | 0.0015 | 0.0000 | 0.0000 |
| Asgardaeota | 0.0000 | 0.0000 | 0.0000 | 0.0000 | 0.0009 | 0.0011 |
| Entotheonellaeota | 0.0003 | 0.0018 | 0.0000 | 0.0000 | 0.0000 | 0.0016 |
| Arthropoda | 0.0000 | 0.0018 | 0.0000 | 0.0013 | 0.0000 | 0.0000 |
| Phragmoplastophyta | 0.0000 | 0.0000 | 0.0000 | 0.0020 | 0.0000 | 0.0004 |
| Euglenozoa | 0.0000 | 0.0000 | 0.0000 | 0.0000 | 0.0000 | 0.0027 |
| TA06 | 0.0000 | 0.0000 | 0.0000 | 0.0000 | 0.0010 | 0.0000 |
| GAL15 | 0.0009 | 0.0000 | 0.0000 | 0.0000 | 0.0004 | 0.0000 |
| Ciliophora | 0.0000 | 0.0014 | 0.0000 | 0.0015 | 0.0000 | 0.0011 |
| Protalveolata | 0.0000 | 0.0014 | 0.0000 | 0.0000 | 0.0000 | 0.0000 |
| Peronosporomycetes | 0.0000 | 0.0000 | 0.0000 | 0.0010 | 0.0000 | 0.0000 |
| Microsporidia | 0.0000 | 0.0000 | 0.0000 | 0.0010 | 0.0000 | 0.0000 |
| Dadabacteria | 0.0003 | 0.0000 | 0.0000 | 0.0000 | 0.0006 | 0.0000 |
| LCP-89 | 0.0000 | 0.0000 | 0.0000 | 0.0000 | 0.0003 | 0.0004 |
| Pavlovophyceae | 0.0000 | 0.0000 | 0.0000 | 0.0005 | 0.0000 | 0.0000 |
| Cryptomycota | 0.0000 | 0.0000 | 0.0000 | 0.0005 | 0.0000 | 0.0000 |
